# Reward and punishment: investigating cortico-bulbar excitability to disclose the value of goods

**DOI:** 10.3389/fpsyg.2013.00039

**Published:** 2013-02-05

**Authors:** Carmelo M. Vicario, Ada Kritikos, Alessio Avenanti, Robert Rafal

**Affiliations:** ^1^School of Psychology, University of QueenslandBrisbane, QLD, Australia; ^2^Faculty of Motor Science, University of PalermoPalermo, Italy; ^3^Department of Psychology, University of Bologna “Alma Mater Studiorum,”Bologna, Italy; ^4^Centro di Studi e Ricerche in Neuroscienze Cognitive, Polo Scientifico-Didattico di CesenaCesena, Italy; ^5^Istituto Di Ricovero e Cura a Carattere Scientifico, Fondazione Santa LuciaRoma, Italy; ^6^School of Psychology, Univerisity of WalesBangor, UK

A continuing challenge for neuroscientists is to develop new conceptual tools and methodologies for understanding, predicting, and modeling the influences of rewarding/punishing outcomes on human behavior and decision-making. Reinforcement shapes behaviors from the most primitive (fight/flight, ingest/regurgitate, approach/avoid) to complex (buy/sell). Understanding the neural processes underlying reinforcement is critical for understanding economic and social decision-making. Moreover, comprehension of de-ranged processing and responses to reinforcing stimuli is crucial across a range of psychology fields and society as a whole, including psychiatric and neurological illness, eating disorders, criminality, and sociopathy (Vicario and Crescentini, 2012).

Neuroimaging methods have provided important information on the neural network underlying reward processing. Studies have shown that the reward value (O'Doherty et al., 2000) and the subjective pleasantness (Kringelbach et al., 2003) of food and non-food stimuli are reflected in the activity of orbitofrontal cortex (OFC). This region has been implicated in computing the hedonic value of food and in response to reward predicting signals in humans (Anderson et al., 2003). The OFC is part of a widespread network that includes also several subcortical regions such as hypothalamus, amygdala, dopaminergic midbrain, as well as parts of the basal ganglia including the ventral and dorsal striatum (Kringelbach, 2005). The network includes also cortical regions such as the insula and anterior cingulate cortex (ACC) which are reciprocally connected to the OFC and to hypothalamic and brainstem pathways mediating autonomic and visceral control (Freedman et al., 2000).

Dysregulation of this network could be responsible for several typologies of eating disorders such as anorexia nervosa (AN) and hyperphagia. Decreases in striatal D2 receptors have been linked to compulsive food intake in obese rodents (Johnson and Kenny, 2010) and with decreased metabolic activity in OFC and ACC in obese humans (Tremblay and Schultz, 1999). On the other hand, it was suggested that some changes in dopamine D2 receptor binding in obese individuals may be a consequence rather than the cause of their over-eating (Berridge et al., 2010). Dopaminergic dysfunction, particularly in striatal circuits, might also contribute to altered reward and decreased food ingestion in individuals with AN (Kaye et al., 2009). For example, a seminal imaging study reported that the observation of food pictures by underweight subjects with AN led to altered activity in insula, OFC, and ACC (Frank et al., 2005). The role of dopaminergic alteration corresponding to these midbrain circuits was furthermore demonstrated in a recent work reporting that patients with Parkinson's disease (PD) experienced less motivational arousal for appetitive images compared with individuals of the same age without PD (Shore et al., 2011). By contrast, hyperphagic individuals with Prader–Willi syndrome manifested enhanced motivational arousal for appetitive images in the same paradigm (Hinton et al., 2010).

Modulation of reward circuitry was observed when viewing faces contingent on their sexual attractiveness (Kranz and Ishai, 2006) or by monetary compensation (Urban et al., 2012). For instance, in a decision-making task (O'Doherty et al., 2001) participants had to associate arbitrary stimuli with monetary wins or losses. A dissociation in location of fMRI signals was found such that activity in the medial OFC cortex correlated with wins on single trials, and activity of the lateral OFC cortex correlated with losses on single trials. Finally, reward related circuits for appetitive stimuli are also involved in social evaluation. For example, the insula is a key region in processing disgusting odors (Wicker et al., 2003) but is also modulated when processing social and moral emotions such empathy, guilt, and shame (Wicker et al., 2003; Moll et al., 2005; Lamm and Singer, 2010; Azevedo et al., 2012). For example, observing faces of people expressing disgust activates the anterior insula of the observer similarly to what would occur if the observer was disgusted himself (Wicker et al., 2003; Jabbi et al., 2007). Moreover, Sanfey et al. (2003) reported insula activation, in participants who received unfair monetary offers. These findings indicate that sensorial and social disgust might share, at least in part, common neural pathways (see also Schaich Borg et al., 2008). In keeping with this proposal, scholars have suggested that higher mental processes, such as those mediating interpersonal and moral disgust, may have originated from the corresponding sensorial pattern (Haidt et al., 1997; Chapman et al., 2009).

While the research reviewed above highlights the usefulness of neuroimaging techniques in functionally localizing the correlates of reward/punishment outcomes, other methodological approaches are needed to address several key issues that are opaque to functional neuroimaging. It should be taken into account that blood oxygen level dependent fMRI alone cannot determine whether neural activity is excitatory or inhibitory in nature, it has low temporal resolution and its signal mainly represents the local field potentials thus reflecting more the local processing of inputs rather than the output signals (Goense and Logothetis, 2008).

To overcome some of these limitations, we suggest an embodied cognition approach for the investigation of the psychobiology of reinforcement. According to previous suggestion (Vicario and Candidi, 2011; Vicario and Ticini, 2012), we propose the hypothesis that somatosensory intra-oral activity might represent a possible somatic marker linking neural processes and motor output along the reward/punishment continuum. There are a number of arguments in support of the view that reward/punishment stimuli may influence the somatosensory intra-oral activity. First, it is well-established that cortico-bulbar motor neurons in rats respond to four basic tastes (Yamamoto et al., 1982). Second, studies have shown that agonists and antagonists of dopamine, a neurotransmitter critically involved in the regulation of the reward system, spontaneously activate neurons in the nucleus tractus solitarius (NTS), dorsal motor nucleus of the vagus (nX), and nucleus nervi hypoglossi (nXI1) (Granata and Woodruff, 1982), cortico-bulbar components actively involved in the regulating feeding behavior (see Simon et al., 2006). More recently, Pilarski et al. (2012) have shown in mice that chronic exposure to nicotine, which affects the activity of the reward system during embryonic development and during the first week of life, also alters neurotransmission and excitability in hypoglossal motoneurons by decreasing the glutamatergic excitatory synaptic input of these neurons. These findings hint at the possible functional link between the neural cortico-bulbar representation of intraoral muscles and basic rewards processing. Notably, previous work using psychophysiological assessment in humans has provided indirect support for such link which may extend to non-food reinforcers. For example, using peripheral electromyography (EMG) Chapman et al. (2009) showed that activity of the levator labii—a muscle involved in the facial expression of disgust—increased not only when drinking unpleasant liquids, but also when watching disgusting pictures of contaminants, and when exposed to unfair treatment in an economic game (i.e., during moral disgust). In a similar vein, Gal (2012) has shown that salivary secretion is stimulated even by material rewards such as money or sport cars, similar to what happens with physiological hunger (refer to the Table 1 for details). However, to date, no studies have directly investigated whether food and non-food reinforcement stimuli, along the rewarding-punishing continuum, modulate the cortical representation of intra-oral muscles, although a recent work has shown that combined odor and tastant stimulation (i.e., flavor of lemon) can increase motor-evoked potentials (MEPs) amplitude from the submental muscles during swallowing (Wahab et al., 2010).

**Table 1:** **Experimental evidence in support of relation between cortico-bulbar excitability and reward/punishment outcomes**.

	**Orality and its link to reward/punishment outcomes**	
**Cue**	**Reward**	**Punishment**
Monetary	(Gal, 2012) Exposure to money stimulates salivary response.	
Food	(Yamamoto et al., 1982) Cortico-bulbar motor neurons in rats respond to four basic tastes.	
	(Wahab et al., 2010) Odor and tastant stimulation (i.e., flavor of lemon) increase cortical excitability of submental muscles during swallowing.	
Luxury good	(Gal, 2012) Exposure to sports cars stimulates salivary response.	
Morality		(Chapman et al., 2009) Unfair treatment in an economic game activates the “levator labii” muscle as well as sensorial disgust.
Drug	(Hajiha et al., 2009) Opioid dose-related suppression of genioglossal muscle activity.	
	Pilarski et al., 2012 Chronic exposure to nicotine, alters neurotransmission and excitability in hypoglossal motoneurons.	

We propose that reinforcement stimuli along the rewarding-punishing continuum can be mapped in a specific somatotopic pattern of intra-oral muscles. This pattern can be investigated at a cortical level using specific neurophysiological assessment that allows to measure changes in the excitability of tongue representations in the primary motor cortex and cortico-bulbar system. Transcranial magnetic stimulation (TMS) represents an extraordinary method to non-invasively stimulate the human motor cortex and assess motor excitability of specific body parts, by recording MEPs from target muscles. Notably, changes in motor excitability as assessed by means of TMS-induced MEPs can occur in the absence of peripheral changes in EMG. Thus, this method is in principle more sensitive than peripheral EMG and can provide more direct information about neural processing at a cortical level. Importantly, this method offers high temporal resolution, can disambiguate between inhibitory and excitatory neural activity by assessing the amplitude of MEPs and has been effectively used to explore sensorimotor functions (Hallett, 2007; Avenanti et al., 2012a,b) as well as social cognition (Schütz-Bosbach et al., 2009; Candidi et al., 2010; Avenanti et al., 2012c).

Relevant to the present proposal, TMS studies have already shown that perception of emotional stimuli modulates the excitability of upper-limb motor representations. For example, watching emotionally arousing pictures (i.e., sexual scenes or images depicting aggressions or disgusting situations) brings about a general increase of motor excitability (Hajcak et al., 2007; van Loon et al., 2010; Borgomaneri et al., 2012), in keeping with the notion that emotion prime the body for action (Frijda, 2009). On the other hand, different types of salient stimuli may also induce a fast reduction of motor excitability. For example, visual flashes (Cantello et al., 2000) or unexpected loud auditory stimuli (Furubayashi et al., 2000) as well as suddenly approaching objects (Makin et al., 2009) or sounds (Serino et al., 2009; Avenanti et al., 2012a) occurring near the body (i.e., within the peripersonal space; Serino et al., 2011) are typically associated to short-latency motor inhibition. Moreover, reduced motor excitability is found during administration of noxious stimuli to a specific body part (Farina et al., 2001; Urban et al., 2004) or when observing pain stimuli on the same body of others (Minio-Paluello et al., 2006; Avenanti et al., 2009). All these findings have been interpreted as reflecting specific approaching/avoidance and freezing upper-limb motor responses to emotionally salient stimuli. The fine-grained topographic features of such motor modulations hint at the possibility that reinforcement stimuli, and in particular food, may specifically modulate neural activity within tongue motor cortical representations.

When applied to the primary orofacial motor cortex, TMS can be used to monitor changes in the excitability of the cortico-bulbar representations of tongue or lip muscles (Muellbacher et al., 1994) with a relatively high temporal resolution. This can be done by measuring tongue MEPs elicited by TMS from the cortico-bulbar pathway under various experimental conditions. Previous TMS studies show that the amplitude of tongue MEPs is modulated by motor training (e.g., repeated tongue protrusion), pain, and language-related processing (i.e., speech listening or viewing) (Fadiga et al., 2002; Watkins et al., 2003; Boudreau et al., 2007; Sato et al., 2010). The excitability of tongue motor representations may be modulated directly or indirectly (i.e., via the premotor cortex) by the outputs originating from the reward system network and possibly conveyed through the axonal pathways linking the tongue with the primary gustatory cortex. We speculate that food, and possibly evolutionarily advanced reinforcers such as monetary compensation or social disgust may impinge on motor representations of intraoral muscles in the cortex. Future studies are needed to directly test the influence of rewards on tongue and lips cortico-bulbar representation.
